# SP-1, a Serine Protease from the Gut Microbiota, Influences Colitis and Drives Intestinal Dysbiosis in Mice

**DOI:** 10.3390/cells10102658

**Published:** 2021-10-05

**Authors:** Aicha Kriaa, Amin Jablaoui, Soufien Rhimi, Souha Soussou, Héla Mkaouar, Vincent Mariaule, Natalia Gruba, Ali Gargouri, Emmanuelle Maguin, Adam Lesner, Moez Rhimi

**Affiliations:** 1 Microbiota Interaction with Human and Animal Team (MIHA), Micalis Institute-UMR1319, AgroParisTech, Université Paris-Saclay, INRAE, F-78350 Jouy-en-Josas, France; aicha.kriaa@inrae.fr (A.K.); amin.jablaoui@inrae.fr (A.J.); soufienrhimi@yahoo.fr (S.R.); souha.soussou.1994@gmail.com (S.S.); hela.mkaouar@inrae.fr (H.M.); vincent.mariaule@inrae.fr (V.M.); emmanuelle.maguin@inare.fr (E.M.); 2 Faculty of Chemistry, University of Gdansk, Uniwersytet Gdanski, Chemistry, Wita Stwosza 63, PL80-308 Gdansk, Poland; natalia.gruba@ug.edu.pl (N.G.); adam.lesner@ug.edu.pl (A.L.); 3 Laboratory of Molecular Biotechnology of Eukaryotes, Center of Biotechnology of Sfax, University of Sfax, Sfax Bp ‘1177’ 3018, Tunisia; faouzi.gargouri@cbs.rnrt.tn

**Keywords:** inflammatory bowel diseases, gut microbiota, serine proteases, microbiome, holobiont

## Abstract

Increased protease activity has been linked to the pathogenesis of IBD. While most studies have been focusing on host proteases in gut inflammation, it remains unclear how to address the potential contribution of their bacterial counterparts. In the present study, we report a functional characterization of a newly identified serine protease, SP-1, from the human gut microbiota. The serine protease repertoire of gut *Clostridium* was first explored, and the specificity of SP-1 was analyzed using a combinatorial chemistry method. Combining in vitro analyses and a mouse model of colitis, we show that oral administration of recombinant bacteria secreting SP-1 (i) compromises the epithelial barrier, (ii) alters the microbial community, and (ii) exacerbates colitis. These findings suggest that gut microbial protease activity may constitute a valuable contributor to IBD and could, therefore, represent a promising target for the treatment of the disease.

## 1. Introduction

Inflammatory bowel disease (IBD) is a chronic and potentially debilitating disorder of a multifactorial etiology [1,2,3]. Several factors have been involved in the disease, including genetic susceptibility, a dysregulated immune response, and gut microbiota alterations [4,5]. Patients with IBD exhibit reduced levels of specific taxa, including *Lactobacillus* and *Bifidobacterium*, while members of the Enterobacteriaceae family, such as *Escherichia coli,* increase [6,7,8]. Significant enrichment of *Clostridium* spp. species, mainly *Clostridium ramosum*, was also detected in mice with colitis and patients with IBD [9,10,11]. *C. ramosum* is a common enteric anaerobe but can be occasionally isolated as an opportunistic pathogen [12]. Some strains display proteolytic activities that may promote intestinal colonization, allowing bacteria to escape mucosal defenses [13,14]. Proteases play many roles in regulating key patho/physiological processes in both eukaryotes and prokaryotes [15,16,17,18,19]. In prokaryotes, they are intricately involved in bacterial physiology. They help the bacterium outcompeting and establishing within the gut microbiota and probably resisting the hostile gastrointestinal (GI) environment [20,21]. Note that they may also serve as virulence factors for pathogenic bacteria, disrupt mucosal barriers, and subvert host immune responses [22,23,24,25]. Surprisingly, the role of such microbial proteolytic activities has been scarcely addressed, and the relevance of bacterial protease supplementation on host physiology and microbiota composition is seldom explored. Earlier studies have unveiled significant contributions of bacterial proteases to the proteolysis in the human large intestine [26,27]. Most identified proteases were likely to belong to *Bacteroides*, *Streptococcus,* and *Clostridium* [27]. With regard to GI inflammation, characterized gut microbial proteases include those from *Enterococcus faecalis*, *Helicobacter pylori*, *Escherichia coli,* and non-pathogenic bacterium *Bacillus subtilis* [24,25,28,29,30]. In the present study, we analyze the effect of a putative serine protease from *C. ramosum*, hereinafter denoted as SP-1, on intestinal inflammation. Our analyses show that SP-1 belongs to a distinct group of unexplored protease-producing *Clostridium* and exhibits greater differences with known peptidases. Administered SP-1 increased epithelial permeability, dysregulated the gut microbiota, and worsened inflammation in the dextran sodium sulfate (DSS) mouse model of colitis.

## 2. Materials and Methods

### 2.1. Bacterial Strains and Media

*Clostridium ramosum* DSM1402 strain was grown under anaerobic conditions in 110 medium (Anaerobe systems, Morgan Hill, CA, USA) with 15% rumen fluid. *Escherichia coli* BL21 (DE3) was used as a host for heterologous expression of the candidate gene. *E. coli* cultures were grown in Luria–Bertani (LB) medium (MP Biomedicals, Irvine, CA, USA). Cultures of *Lactococcus lactis* strains (*L. lactis*-WT and *L. lactis*-SP-1) were performed in M17 medium (Oxoid, Thermo-Fisher Scientific Inc., Hampshire, UK) supplemented with 0.5% glucose at 30 °C without shaking.

### 2.2. Amino Acid Sequence Alignment and Phylogenetic Analysis

Protein alignments were performed using ClustalW, and the results were visualized using Espript 3.0 [31]. Conserved residues were highlighted according to specific criteria set by the software (http://espript.ibcp.fr/ESPript/ESPript/esp_userguide.php#line_4, accessed on 1 July 2021). *Clostridium* subtilisin-like protease sequences were retrieved from the public database NCBI. The resulting data were filtered to remove partial and redundant sequences using an internally developed script based on the identity of protease sequences. This reference set was used to look against the human gut metagenomic catalog of ~10 million genes in order to retrieve gut *Clostridium* sequences at a threshold of 90% sequence identity and track phylogenetic relationships [32]. The tree was saved in Newick format and uploaded to the iTOL web server for visualization [33]. The average number of S8 peptidases per analyzed *Clostridium* genome was estimated. Only completely annotated genomes were considered.

### 2.3. Cloning and Plasmids

For the expression of recombinant SP-1 in *E. coli*, the chromosomal DNA was first isolated from a culture of *C. ramosum* DSM1402 using the Wizard^®^ Genomic DNA Purification kit (Promega). The gene encoding the mature polypeptide of SP-1 was amplified using the specific primers, SP-1/NcoI-F 5′GGCCATGGCAAATAATAATTATGAGCTTGG3′ and SP-1/XhoI-R 5′ACTAGTCTCGAGTTATTCTCGTCCATGCCAGCC3′. Restriction sites (underlined) for the endonucleases NcoI and XhoI were used for cloning. The PCR product was digested, purified, and cloned into pETM-11, thereby fusing a hexa-histidine-tag (His 6-tag) sequence at the N-terminus of full-length sp-1. This plasmid was transformed into the bacterial strain BL21 (DE3) using standard procedures, and constructions were checked by DNA sequencing.

For the expression and secretion of SP-1 in *L. lactis* NZ9000, the sp-1 gene was amplified by PCR reaction using SP-1/NsiI-F 5′GGATGCAT**CA**AATAATAATTATGAGCTTGG3′ and SP-1/EcorI-R 5′ACTAGTGAATTCTTATTCTCGTCCATGCCAGCAGTT3′. Primers were designed to harbor one restriction site at each end. The NsiI site is underlined in which CA (in boldface type) was added just after the restriction site to ensure the cloning of sp-1 in the same reading frame as SPUsp. pSEC:LEISS was then used to clone the sp-1 gene under the transcriptional control of lactococcal nisin-inducible promoter PnisA [34]. In the resulted plasmid pSec-SP-1, the sp-1 gene was fused in frame with a DNA fragment encoding for the ribosome-binding site (rbs) and the signal peptide SPUsp, the main *L. lactis*-secreted protein [35].

### 2.4. Expression and Purification of Recombinant SP-1

Following overnight pre-culture, recombinant *E. coli* was grown in Luria broth to OD600 ∼ 0.6. Gene expression was induced with the addition of 0.5 mM IPTG (Sigma-Aldrich) at 25 °C. Recombinant strains were maintained with kanamycin (25 µg/mL). A total of 3 h after induction, bacteria were harvested by centrifugation at 6000 rpm for 20 min at 4 °C. Cell pellets were then washed, homogenized in 20 mM Tris, 300 mM NaCl, pH 8, and incubated for 1 h on ice in the presence of 10 mM MgCl_2_ and 200 U benzonase (Novagen^®^). Following sonication at 4 °C for 1 min (three cycles of 10 s on followed by 10 s off intervals), the lysate was centrifuged at 12,000 rpm for 20 min at 4 °C. The resulting supernatant with the soluble fraction was collected to analyze SP-1 activity. Purification was achieved by affinity chromatography using 1 mL HiTrap chelating HP column (GE Healthcare) charged with Ni^2+^ ions on the ÄKTA Purifier FPLC system (Amersham Pharmacia Biotech) and equilibrated with 20 mM Tris, pH 8 buffer supplemented with 300 mM NaCl. Proteins were eluted using a linear imidazole gradient ranging from 0 to 500 mM. Fractions were pooled, and proteins were checked by SDS-polyacrylamide gel electrophoresis (SDS-PAGE).

For induction of SP-1 production in *L. lactis*, exponential growth cultures (OD600 = 0.6) of *L. lactis* strains were treated for 3 h with nisin (1 ng/mL, Sigma-Aldrich) to induce protease expression. Recombinant strains were maintained with chloramphenicol (10 µg/mL). After nisin induction, bacteria were centrifuged at 4500 rpm for 20 min at room temperature, washed, and suspended in the corresponding volume of sterile phosphate-buffered saline (PBS) to obtain 5 × 10^9^ CFU/mL for oral treatment in mice. Cultures were routinely tested for purity.

### 2.5. Western Blotting and Kinetic Constant Determination

Equal amounts of protein were run on 15% SDS-PAGE, transferred to PVDF (BDH), and immunodetected by anti-histidine (Sigma-Aldrich) primary antibody followed by peroxidase-coupled secondary antibody (Sigma-Aldrich). Proteins were visualized by enhanced chemiluminescence (Chemidoc, Bio-Rad). For kinetic constant determination, SP-1 (20 nM) was incubated at room temperature, in a microtiter plate, with assay buffer (20 mM Tris, 300 mM NaCl, 1 mM CaCl_2_, pH 8) and varying concentrations (0.05–2 mM) of synthetic substrates in a final volume of 100 μL. Activity measurements were performed using a plate reader (Synergy™ 2 Multi-Mode Microplate Reader, BioTek^®^) set at (410 nm), for released ANB-NH_2_, or (Excitation: 360 nm, Emission: 460 nm), for the fluorogenic substrates. Enzyme-free reactions were used as negative controls. All experiments were performed in triplicate. Standard curves were used to calculate the rate of product generation. Data were processed by non-linear regression analysis with GraphPad Prism 8.0, GraphPad Software (San Diego, CA, USA) and globally fitted to the Michaelis–Menten equation.

### 2.6. Peptide Library Preparation

To screen the P1 substrate residues, we used 19 libraries with the general formula: ABZ-P4-P3-P2-P1-ANB-NH_2_, where the P1 residue in each library was defined, while the P4-P2 positions were occupied by a mixture of 19 proteinogenic amino acid residues. Incubation of libraries with purified SP-1 allowed the selection of Arg as the optimal P1 residue. Next, a peptide library with a fixed Arg residue at P1 was synthesized by the mixing-portioning method [36]. A total of 11.4 g of the solid support (Tenta-Gel S RAM) was used for the ABZ-P4-P3-P2-Arg-ANB-NH_2_ library preparation (wherein positions P4, P3, P2, the set of proteinogenic amino acid residues, except Cys, was present). The synthesis was initiated by the deprotection of the amino groups of the resin with 20% piperidine in NMP (1-methyl-2-pyrrolidone), then the resin-bound amino group was acylated with 5-amino-2-nitrobenzoic acid using the N,N,N’,N’-tetramethyl-O-(benzotriazol-1-yl)uranium tetrafluoroborate (TBTU)/4-dimethylaminopyridine (DMAP). The resin was washed twice with N-methylmorpholine. Next, two equivalents of ANB and two equivalents of TBTU/DMAP were dissolved in DMF and added to the resin. Upon 30 s also four equivalents of N,N-diisopropylethylamine (DIPEA) were added. The reaction was continued for 3 h. To ensure a complete resin substitution, the procedure was repeated three times. The first Fmoc-protected amino acid was attached to ANB by POCl3/pyridine method [37]. After deprotection for all subsequent Fmoc-protected amino acids, DIPCI/HOBt coupling system was applied using a three-fold excess.

The obtained peptides were cleaved from the resin using a trifluoroacetic acid (TFA)/phenol/ triisopropylsilane/H_2_O mixture (88:5:2:5, *v/v*) [38]. The purity of the synthesized peptides was checked on RP-HPLC Jasco LC System (Jasco, Japan) equipped with Supelco Wide Pore C8 column (8 × 250 mm) and ultraviolet-visible (UV-VIS, 226 nm) and fluorescent detectors (excitation 320 nm, emission 450 nm). A linear gradient from 10% to 90% B within 40 min was applied (A: 0.1% TFA in water; B: 80% acetonitrile in A). Mass spectra analysis of synthesized peptides was performed using a Biflex III MALDI TOF mass spectrometer (Bruker, Germany) using α-cyano-4-hydroxycynnamic acid (CCA) or 2.5-dihydroxybenzoic acid (DHB) as a matrix.

### 2.7. Animals

All mice used in this study were male C57BL/6 and 6–8 weeks old. They were purchased from Janvier (Janvier, le Genest Saint Isle, France) then cohoused for a week before inclusion in the study. All procedures were performed according to the European Community Rules and approved by the Animal Care Committee (C2E−45 COMETHEA) with authorization number APAFiS #16989.

### 2.8. Assessment of Mild Inflammation and Study Design

Mice received DSS (1.5% *w*/*v*, 36–50 kDa, MP Biomedicals, CA) supplemented in the drinking water for 7 days. A total of 5 × 10^9^ CFU of either *L. lactis*-WT or *L. lactis*-SP-1 were administered via oral route into mice for the whole period of DSS treatment. Changes in body weight, stool consistency, presence of gross blood in feces were evaluated daily and scored for each mouse over the experimental period. The disease activity index (DAI) was calculated as previously reported (Table 1) [39]. At the end of the experimental period, mice were sacrificed, and the entire colon was excised for the measurement of inflammatory parameters.

### 2.9. MPO Activity Measurement

MPO activity was assessed as previously described [40]. Briefly, a colon segment was homogenized (Precellys Evolution Homogenizer, Bertin Instruments) in 50 mM phosphate buffer, pH 6 at 4 °C. The colon suspension was 10-fold-diluted in 50 mM phosphate buffer containing 0.5% hexadecyltrimethyl ammonium bromide (Sigma-Aldrich). After sonication for 10 s and subjection to 3 freeze-thaw cycles, samples were centrifuged at 14,000 rpm for 10 min. A total of 10 μL of each supernatant was then added to 290 μL of 50 mM phosphate buffer, pH 6 containing 0.167 mg mL^−1^ of o-dianisidine dihydrochloride (Sigma-Aldrich) and 0.0005% H_2_O_2_. Changes in absorbance at 460 nm were measured over 5 min. MPO activity was determined by comparison to a standard MPO curve.

### 2.10. Histological Evaluation and Scoring

Hematoxylin and eosin (H&E) colonic tissue sections were prepared at the @BRIDGe platform (INRA, Jouy-en-Josas) by incubation with 4% (*v/v*) buffered formalin and 70% (*v/v*) alcohol and then embedded in paraffin. Tissue sections of the distal colon were prepared, stained with H&E, and further analyzed. The severity of colonic histological damage was scored in a blinded fashion to prevent observer bias, as follows: neutrophil infiltration (0–2), lymphocyte infiltration (0–2), erosion (0–3), and crypt loss (0–2).

### 2.11. Cell Cultures, SP-1 Treatment and Intestinal Permeability Assay

The epithelial barrier model was achieved using the human colon epithelial cell line Caco-2 clone TC7 [41]. Caco-2 cells were cultured in Dulbecco’s modified eagle medium (DMEM, Life Technologies, Grand Island, NE, USA) supplemented with 10% fetal calf serum (FBS), 100 U mL^−1^ penicillin, and 100 μg mL^−1^ streptomycin at 37 °C in a 5% CO_2_ incubator. For the permeability assay, cells were seeded on a Transwell^®^ insert filter (0.4 μm pore size, 12 mm diameter polycarbonate membrane; Costar, Corning Life Science, Kennebunk, USA) at 10^5^ cells per cm^2^. Once the optimal transepithelial electrical resistance has reached (TEER ≥ 1800 Ω/cm^2^ measured using a multicellular electrical resistance system; Millipore, Billerica, MA, USA), fresh medium was added, and the cells were then treated (or not) in the apical side with increased concentrations of recombinant SP-1 (0.1 µg/mL, 0.5 µg/mL, and 1 µg/mL). The results were expressed in the percentage of TEER for each cell culture condition. Three different experiments were performed, including duplicates of each condition.

Intestinal permeability was assessed 7 days after DSS treatment using fluorescein isothiocyanate (FITC)-dextran (4 kDa, Sigma-Aldrich). Mice were orally gavaged with FITC-dextran, and serum samples were collected after 3 h.

### 2.12. Cytokine Quantification

Colonic tissues were homogenized in lysis buffer (50 mM Tris pH 7.5, 500 mM NaCl, 2 mM EDTA, 1% Triton X-100 (*v/v*), 0.5% sodium deoxycholate, 0.1% SDS) containing a protease inhibitor mixture. Samples were sonicated for 2 min, then centrifuged for 10 min at 14,000 rpm, and the resulting supernatants were frozen at −80 °C until use. Cytokine levels in colon tissues were determined by immunoassay using a U-plex kit (MSD) according to the manufacturer’s instructions.

### 2.13. Fecal Protease Activity Measurement

For each biological sample, 50 mg of feces was thawed and homogenized in 1.5 mL of assay buffer (20 mM Tris, 300 mM NaCl, pH 8). After centrifugation (15 min, 5000 rpm, 4 °C), pellets were discarded, and supernatants were filtered using size syringe filters (0.8 µm, Nalgene). Supernatants were then used for protease activity measurements by estimating the amount of chromogenic/fluorogenic compound released after proteolytic cleavage. Under operating conditions, the reaction mixture containing 20 µL of fecal water sample at a suitable dilution (100 µg), 160 µL of reaction buffer (20 mM Tris, 300 mM NaCl, 1 mM CaCl_2_ pH 8), and 20 µL of the appropriate substrate to a final volume of 200 µL was incubated during 30 min at room temperature. For casein, the reaction was stopped by adding 100 µL of 10% (*w/v*) trichloroacetic acid (TCA, Sigma-Aldrich). For fecal protease activity profiling, we used different substrates listed in Table 2. We used a serine protease inhibitor, PMSF (Phenylmethylsulfonyl fluoride, 1 mM), for the inhibition assay. Protein concentration was determined using nanodrop (Labtech). One unit of protease activity was defined as the amount of protease catalyzing the formation of 1 μmol of substrate per min under the above experimental conditions. Data are shown as a fold increase in activity detected in control samples.

### 2.14. Microbiota Analysis

The total DNA was extracted from fecal samples using guanidium thiocyanate and the mechanical bead-beating disruption method as previously described [42]. Extracted DNA was amplified using primer pairs designed for V3–V4 fragments, F343 5′CTTTCCCTACACGACGCTCTTCCGATCTACGGRAGGCAGCAG′3 and R784 5′GGAGTTCAGACGTGTGCTCTTCCGATCTTACCAGGGTATCTAATCCT′3. The amplicon lengths were about 450 bp (depending on the species). Given that MiSeq sequencing enables paired 250 bp reads, the ends of each read overlap and can be stitched together to generate extremely high-quality, full-length reads covering the entire V3-V4 region. Single multiplexing was performed using a homemade 6 bp index, which was added during a second PCR with 12 cycles using the forward primer 5′AATGATACGGCGACCACCGAGATCTACACTCTTTCCCTACACGAC3′ and the modified reverse primer 5′CAAGCAGAAGACGGCATACGAGAT-index-GTGACTGGAGTTCAGACGTGT′3. The resulting PCR products were purified and loaded onto the Illumina MiSeq cartridge according to the manufacturer’s instructions. The quality of the run was checked internally using PhiX. Raw sequences were analyzed using the bioinformatics pipeline FROGS (Find Rapidly OTU with Galaxy Solution) [43]. Sequences were quality-filtered and clustered into 97% similarity operational taxonomic units (OTUs) using a cut-off value of 0.03, then classified against the Silva Ribosomal Database using a naive Bayesian approach with an 80% confidence threshold. The final data set yielded 12803 OTUs. Curated OTU sequence data were converted to relative abundance ± SEM. To investigate diversities within-and between-samples (α- and β-, respectively), all samples were rarefied to the same depth before analysis with the phyloseq R package. Shannon index was used to calculate alpha diversity. Differences in phyla, families, and genera were assessed with Kruskal–Wallis test, followed by Dunn’s test [44]. Benjamini–Hochberg corrections (BH) were used to avoid false positives (significance threshold = 0.05) [45]. Significant differences between studied groups were assessed using ANOVA followed by Tukey’s test for pairwise comparison. We used a permutational analysis of variance (PERMANOVA) to test the significance of group differences. Significance was checked using Adonis statistical tests (vegan package of R) to evaluate the distances at 9999 permutations between groups.

### 2.15. Correlation Analyses

Pairwise correlations between each specific genera and host parameters were calculated using Spearman’s nonparametric rank correlation coefficient. We used R 3·3·1, the corrplot and Hmisc package to produce the Spearman correlations matrix, and the Rhea scripts pipeline to perform statistical analysis of the microbiota data [46].

### 2.16. Statistical Analyses

All experiments were performed at least twice with duplicate repeated measures. The results are expressed as means ± SEM. For statistical analysis, GraphPad Prism 8.0 was used. Differences in measured inflammatory parameters between each group were assessed using the Kruskal–Wallis test followed by the Dunn test for multiple comparison test. For inhibition assays, data were analyzed using the Mann–Whitney test to compare the proteolytic activity with and without PMSF. Statistical significance is indicated as * *p* < 0.05, ** *p* < 0.01, and *** *p* < 0.001. The difference was considered significant when the *p*-value was less than 0.05.

## 3. Results

### 3.1. SP-1 Shares Conserved Sequence Features with Subtilisin Despite Overall Dissimilarity

**Figure 1:**
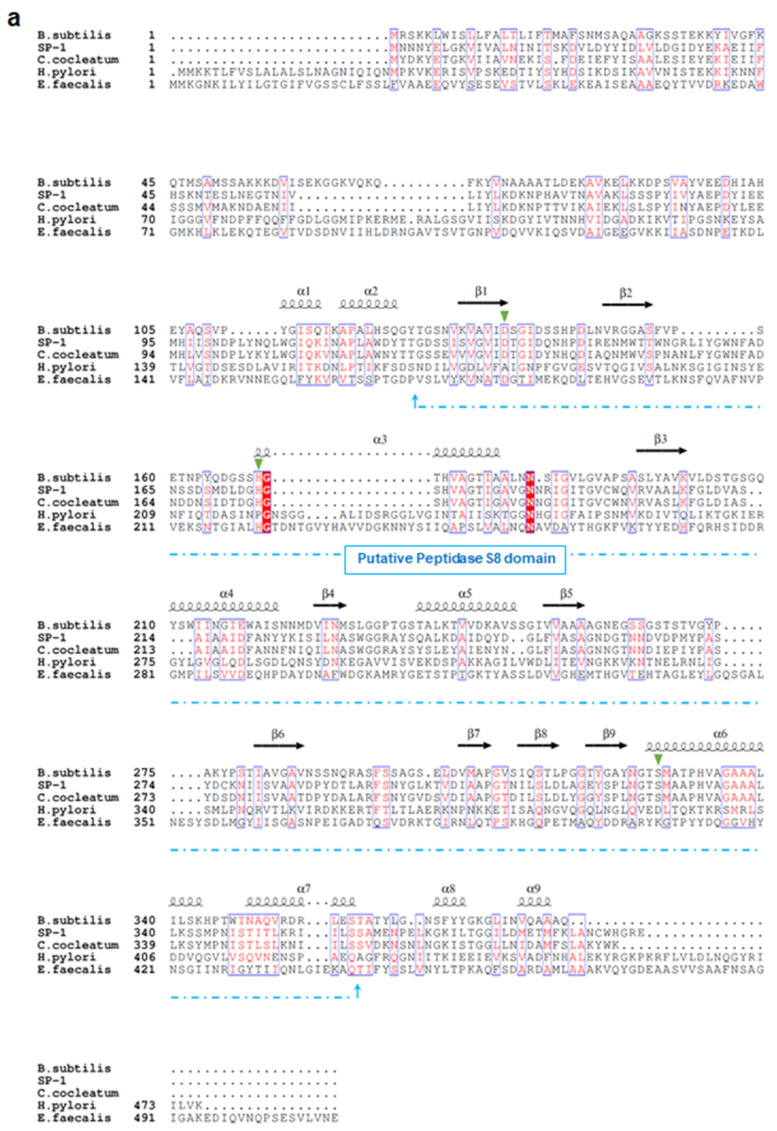

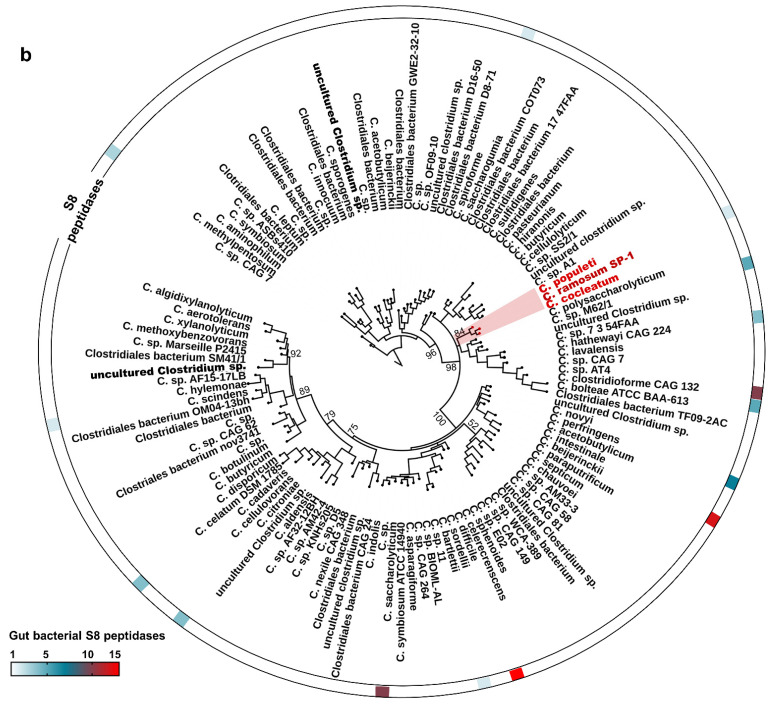
Sequence analysis and phylogeny of *Clostridium*-producing proteases. (**a**) Structure-based sequence alignment of SP-1 and other selected bacterial proteases (UniProt accession numbers: E1S7U4, protease from *H. pylori;* Q833V7, gelatinase from *E. faecalis;* A0A1I0FUC9, protease from *C. cocleatum* and P04189, Subtilisin from *B. subtilis*). The structural elements shown above the alignment were generated using the Subtilisin structure (PDB ID: 6O44). Invariant residues between sequences are typed red on a white background, and conserved residues are shown as white letters on a red background. Green triangles represent conserved amino acids from the catalytic triad, Asp134, His175, and Ser327. (**b**) Distribution of subtilisin-like proteases across gut *Clostridium* species. *Clostridium* sequences with >40% identity to SP-1 are clustered into a subclade highlighted in red. Branch labels show bootstrap values. Outer tracks show the numbers of genes from each S8 subtilisin-like protease in each genome.

Sequence analysis demonstrates that SP-1 displays the highest identities with serine proteases belonging to S8 serine peptidases, including mainly those from *Clostridium cocleatum* (69%), *Coprobacillus cateniformis* (69%), *Anaerotruncus massiliensis* (62%), and *Methanobacterium* sp. (45%). Interestingly, SP-1 amino acid sequence inspection revealed that it displays very low identity values with those from gut species that have been associated with the disease, including *E. faecalis* (gelatinase gelE,8%), *H. pylori* (HtrA,10%), and *B. subtilis* (Subtilisin,26%) (Figure 1a). Subsequent structure-based sequence alignment showed that SP-1 bears a typical peptidase family S8 conserved domain, with a predicted catalytic triad (Asp134, His175, and Ser327). To go further than mere classification, we investigated the *Clostridium subtilisin*-like repertoire in gut bacterial genomes. This analysis resulted in the identification of 117 putative subtilisin-like sequences from the human gut microbiota (Figure 1b). Both *Clostridium* pathogens and commensals encode putative subtilisin-like proteases. The number of these peptidase genes varied across sequenced *Clostridium* genomes (Figure 1b). Note that SP-1, which mapped to a distinct cluster, shared only 40% sequence identity with a putative uncharacterized subtilisin-like protease from *Clostridium populeti* and 69% with that from *Clostridium cocleatum* (Figure 1b). Overall, the low sequence identity and distant phylogenetic relationship with other gut subtilisin-like peptidases suggest that SP-1 is a novel bacterial serine protease.

### 3.2. Purification and Steady-State Kinetic Analysis of SP-1

**Figure 2:**
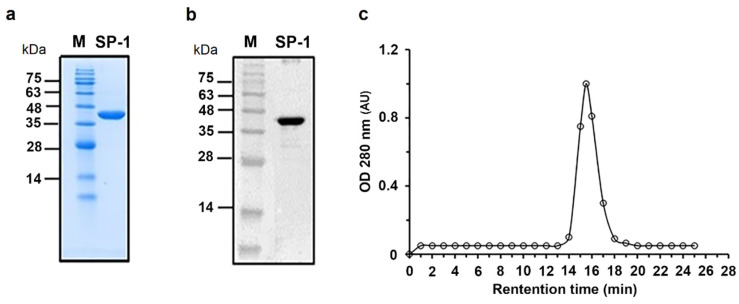
Purification and western blotting of recombinant SP-1. (**a**) Coomassie blue staining of an SDS-PAGE gel. SP-1 is represented by one single band with a molecular mass of 42 kDa. Lane M protein marker (molecular mass in kilodaltons). (**b**) Western blot analysis of purified SP-1 with anti-His tag antibody. (**c**) Size exclusion chromatography analysis of purified SP-1 (retention time, RT, 15 min) using protein markers of 669 kDa (RT, 8.9 min), 440 kDa (RT, 11.3 min), 158 kDa (RT, 13.2 min), 75 kDa (RT, 14.1 min), 44 kDa (RT, 15.3 min) and 29 kDa (RT, 16.9 min).

The gene encoding SP-1 from *C. ramosum* was cloned under control of T7 promoter, in frame with an N-terminal hexa-histidine affinity purification tag, and successfully overexpressed in *E. coli* BL21 (DE3). After a one-step purification procedure, we obtained a soluble recombinant SP-1 with purity ≥ 95%, as shown by SDS-PAGE (Figure 2a), with a purification yield of 1.5 mg of purified protein per liter of bacterial culture. Western blotting using a primary antibody against the hexa-histidine tag showed the presence of a single band with a molecular weight of nearly 42 kDa close to the theoretical mass of SP-1 (Figure 2b). Analysis of the purified protein by size exclusion chromatography showed a single elution peak with an apparent molecular mass of 42 kDa (Figure 2c).

Having suggested that SP-1 is a potential subtilase, we further characterized the enzymatic activity and determined its steady-state kinetic constants. Comparison of the catalytic efficiency for potential target substrates showed that SP-1 exhibits a much higher preference for Arg at position P1 relative to Lys (Table 3). Lower catalytic efficiencies were detected against other substrates (Table 3).

### 3.3. Novel Substrate for SP-1

**Figure 3:**
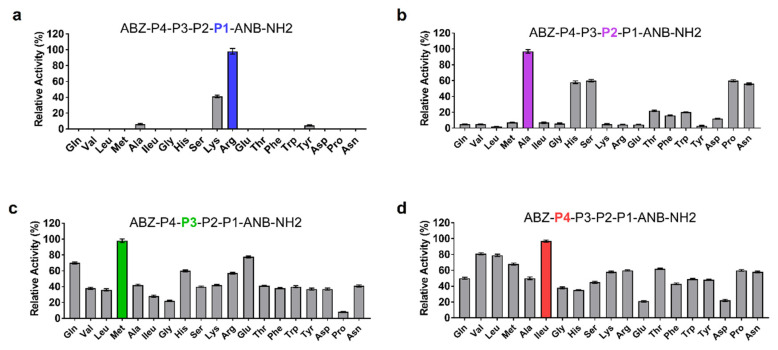
Results of the deconvolution of the ABZ-P4-P3-P2-P1-ANB-NH2 library against SP-1. The x-axis indicates the amino acid fixed at each P position. P1, P2, P3, and P4 correspond to the mixture of 19 amino acid residues. (**a**) P1 position was fixed with the most active residue (**b**) P2, (**c**) P3 and (**d**) P4 profiling were performed in a similar manner. All measurements were performed in triplicate, and the y-axis indicates the activity (mean ± SEM) relative to the mean value of the highest signal detected for each P position.

The molecular basis of SP-1 substrate specificity at P1-P4 positions was analyzed based on an in silico model with the general formula: ABZ-P4-P3-P2-P1-ANB-NH_2_. When screened against SP-1, the sublibrary with Arg in position P1 displayed the highest activity, followed by Lys (Figure 3a). Ala in position P2 appeared to be optimal for SP-1 substrate activity (Figure 3b). Likewise, the presence of Met at position P3 promoted substrate processing (Figure 3c). The last round of deconvolution resulted in the selection of ILeu as an optimal residue at the P4 position (Figure 3d). Significant activity was also displayed with Val and Leu. Finally, a peptide substrate with the sequence ABZ-Ileu-Met-Ala-Arg-ANB-NH_2_ was selected as the most efficient one displaying a high affinity (KM) of 1.91 ± 0.3 × 10**^−4^** M, kcat value of 2.83 ± 0.5 s^−1^, and its catalytic efficiency value reached 1.48 × 10**^4^** M^−1^s^−1^.

### 3.4. Administration of L. Lactis Secreting SP-1 Exacerbates DSS-Induced Colitis

**Figure 4:**
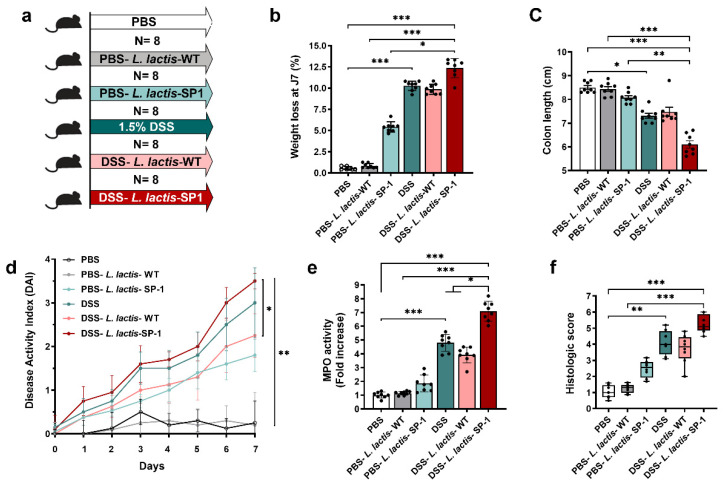
Administration of *L. lactis* secreting SP-1 influences colitis. (**a**) C57BL/6 mice were provided with water or 1.5% DSS-containing water for 7 days (N = 8 in each group). Mice were orally administered with PBS, PBS-*L. lactis*-WT, PBS-*L. lactis*-SP-1, DSS alone, DSS-*L. lactis*-WT or DSS-*L. lactis*- SP-1 (5 × 10^9^ CFU *L. lactis*-WT or SP-1-expressing *L. lactis* were evaluated / day). (**b**) Body weight loss for each group. (**c**–**e**) Mice were euthanized (day 8), and (**c**) colon length, (**d**) disease activity index, and (**e**) colonic MPO activity were measured. (**f**) Histological scores were determined. Data are presented as mean ± SEM from a representative experiment (N = 8 biologically independent animals). Analyzed by Kruskal–Wallis followed by the multi-comparison Dunn’s test. * *p* < 0.05; ** *p* < 0.01 and *** *p* < 0.001.

To investigate the role of SP-1 during inflammation, we experimentally induced mild colonic inflammation through oral DSS administration along with *L. lactis* secreting SP-1 or PBS (Figure 4a). DSS consumption was followed throughout the experiment, and no differences in water consumption were observed between groups (data not shown). As shown in Figure 4b, we found that mice receiving PBS-*L. lactis*-SP-1 showed a significant weight loss in comparison to PBS-*L. lactis*-WT and PBS. In the presence of DSS, SP-1 did not show a significant effect on weight loss and colon length when compared to their control littermates (DSS, DSS-*L. lactis-*WT) (Figure 4b–c). Only in the presence of DSS, SP-1 increased the DAI score (disease activity index, a composite measure of weight loss, stool consistency, and rectal bleeding, Table 1) (Figure 4d). In fact, the DAI was higher in DSS-*L. lactis*-SP-1 mice than DSS-*L. lactis*-WT from the beginning of the DSS challenge and reached statistical significance on day 7 (*p* < 0.05). The notable inflammatory response that accompanies induced colitis was confirmed by a significant increase in myeloperoxidase (MPO) activity (7- and 2-fold higher than PBS-*L. lactis*-WT and DSS-*L. lactis*-WT mice, respectively, *p* < 0.001 and *p* < 0.05), a marker for tissue neutrophil content (Figure 4e). The histological analysis demonstrates a significant increase in the histologic score in mice groups treated with DSS (Figure 4f). Interestingly, the highest score value was obtained in DSS-*L. lactis*-SP-1 (Figure 4f).

### 3.5. SP-1 Increases Gut Inflammation and Fecal Protease Activity in Mice

**Figure 5:**
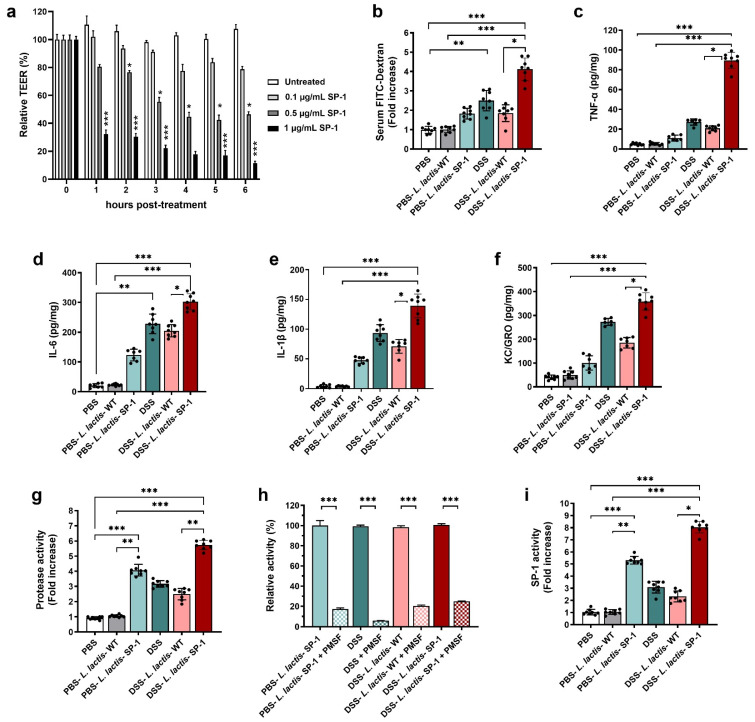
SP-1 supplementation impairs colonic epithelium and increases fecal protease activity. (**a**) Time-dependent means of relative changes (in %) of measured TEER ± SEM in the presence of increasing concentrations of recombinant SP-1 in comparison to untreated cells. (**b**) Epithelial barrier functions were assessed in mice by oral gavage of FITC-dextran on day 7, followed by measuring the FITC-dextran signal in blood after 3 h. (**c**–**f**) Colon tissues were analyzed for the concentrations of pro-inflammatory cytokines. (**g**–**i**) Fecal samples were analyzed for serine protease profiling. (**g**) Total fecal protease activity in each group. (**h**). Relative proteolytic activity without and with pretreatment with PMSF in PBS-*L. lactis*-SP-1, DSS-treated mice, DSS-*L. lactis*-WT and DSS-*L. lactis*-SP-1. The relative activity that reflects the maximal activity was defined as 100%. (**i**) Fecal SP-1 activity in PBS (N = 8), PBS-*L. lactis*-WT (N = 8), PBS-*L. lactis*-SP-1 (N = 8), DSS (N = 8), DSS-*L. lactis*-WT (N = 8) and DSS-*L. lactis*-SP-1 (N = 8). Data are presented as mean ± SEM. Statistical analyses were performed using Kruskal–Wallis, followed by the multi-comparison test of Dunn. * *p* < 0.05; ** *p* < 0.01 and *** *p* < 0.001.

**Figure A1:**
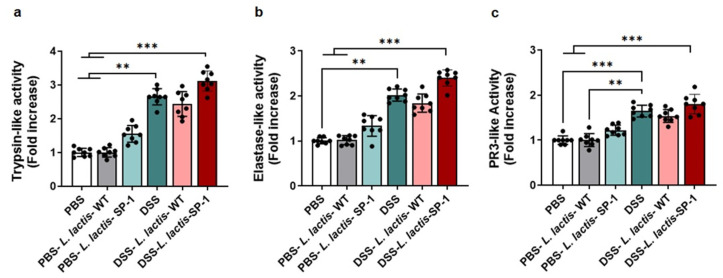
Profiling fecal serine protease activity. (**a**) Fecal trypsin-like activity detected in each group (PBS = 2.25 ± 0.12 U/mg, PBS-*L. lactis*-WT = 2.28 ± 0.14 U/mg, PBS-L. *lactis*-SP-1 = 3.5 ± 0.2 U/mg, DSS = 6.01 ± 0.3 U/mg, DSS-*L. lactis*-WT = 5.48 ± 0.15 U/mg, DSS-*L. lactis*-SP-1 = 7.04 ± 0.2 U/mg). (**b**) Fecal elastase-like activity measurement (PBS = 2.01 ± 0.08 U/mg, PBS-*L. lactis*-WT = 2.06 ± 0.07 U/mg, PBS-*L. lactis-*SP-1 = 2.79 ± 0.2 U/mg, DSS = 4.26 ± 0.13 U/mg, DSS-*L. lactis*-WT = 3.62 ± 0.2 U/mg, DSS-*L. lactis*-SP-1 = 4.81 ± 0.17 U/mg). (**c**) Fecal PR3-like activity measurement (PBS = 1.7 ± 0.09 U/mg, PBS-*L. lactis*-WT = 1.71 ± 0.14 U/mg, PBS-*L. lactis*-SP-1 = 2.05 ± 0.1 U/mg, DSS = 2.81 ± 0.1 U/mg, DSS-*L. lactis*-WT = 2.66 ± 0.15 U/mg, DSS-*L. lactis*-SP-1 = 3.03 ± 0.2 U/mg). The error bars represent the SEM. Data are presented as mean ± SEM. Statistical analyses were performed using Kruskal–Wallis followed by the multi comparison test of Dunn. ** *p* < 0.01 and *** *p* < 0.001.

To explore the relevance of SP-1 activity to barrier dysfunction, we added recombinant SP-1 to the apical side of confluent Caco-2 monolayers. Results showed that SP-1 activity enhanced permeability, as evidenced by a dose-dependent decrease in transepithelial electrical resistance TEER (Figure 5a), in comparison to untreated cells (*p* < 0.05; *p* < 0.001). Based on these results, we examined potential pro-inflammatory effects of SP-1 secretion on the epithelial barrier in DSS-treated mice. To this end, we quantified the permeability by orally administering FITC-dextran to mice and estimating the serum levels. As expected, the diffusion of FITC-dextran through the epithelium was significantly increased in DSS-*L. lactis*-SP-1-treated mice as compared with DSS-*L. lactis*-WT, PBS-*L. lactis*-SP-1, and PBS-*L. lactis*-WT groups (*p* < 0.05; *p* < 0.01; *p* < 0.001, respectively) (Figure 5b). We next examined cytokine production and found that levels of pro-inflammatory TNF-α, IL-6, IL-1β, and neutrophil chemoattractant KC/GRO were significantly increased by 4.2-, 1.4-, 2-, and 1.9-fold, respectively, in DSS-*L. lactis*-SP-1 mice compared with the DSS-*L. lactis*-WT (*p* < 0.001; *p* < 0.05; *p* < 0.01; *p* < 0.05, respectively) (Figure 5c–f). Supporting the hypothesis that uncontrolled protease activities could contribute to colitis, we, therefore, assessed proteolytic activity in fecal samples, which showed 4-fold higher proteolytic activity in PBS-*L. lactis*-SP-1 than PBS-*L. lactis*-WT (*p* < 0.001) (Figure 5g). DSS-*L. lactis*-SP-1 mice showed significantly increased protease activity in comparison to DSS-*L. lactis*-WT (2.5-fold higher, *p* < 0.01) and PBS-*L. lactis*-WT mice (5.7-fold higher, *p* < 0.001) (Figure 5g). In the DSS-*L. lactis*-WT and DSS-*L. lactis*-SP-1 groups, the protease activity was significantly reduced by nearly 79.4% and 75% in the presence of PMSF (*p* < 0.001) (Figure 5h). Seeing that PMSF is a broad-spectrum serine protease inhibitor, we could conclude that serine protease activity increased the most in this context. We also studied the profile of protease subfamilies associated with gut inflammation, including trypsin, neutrophil elastase, and proteinase 3. Our data showed that these activities were slightly increased in DSS-*L. lactis*-SP-1 fecal samples compared with DSS and DSS-*L. lactis*-WT (Figure A1). No statistically significant differences were detected between these groups. Using SP-1 designed substrate, we demonstrated that fecal SP-1 activity was significantly upregulated, a change reflected by 5- and 8-fold increases in PBS-*L. lactis*-SP-1 and DSS-*L. lactis*-SP-1 compared with PBS-*L. lactis*-WT (DSS-*L. lactis*-SP-1 = 11.7 ± 0.4 U/mg, PBS-*L. lactis*-SP-1 = 7.7 ± 0.3 U/mg, PBS-*L. lactis*-WT = 1.47 ± 0.21 U/mg) (Figure 5i).

### 3.6. Alteration of Gut Microbiota and Host Response

**Figure 6:**
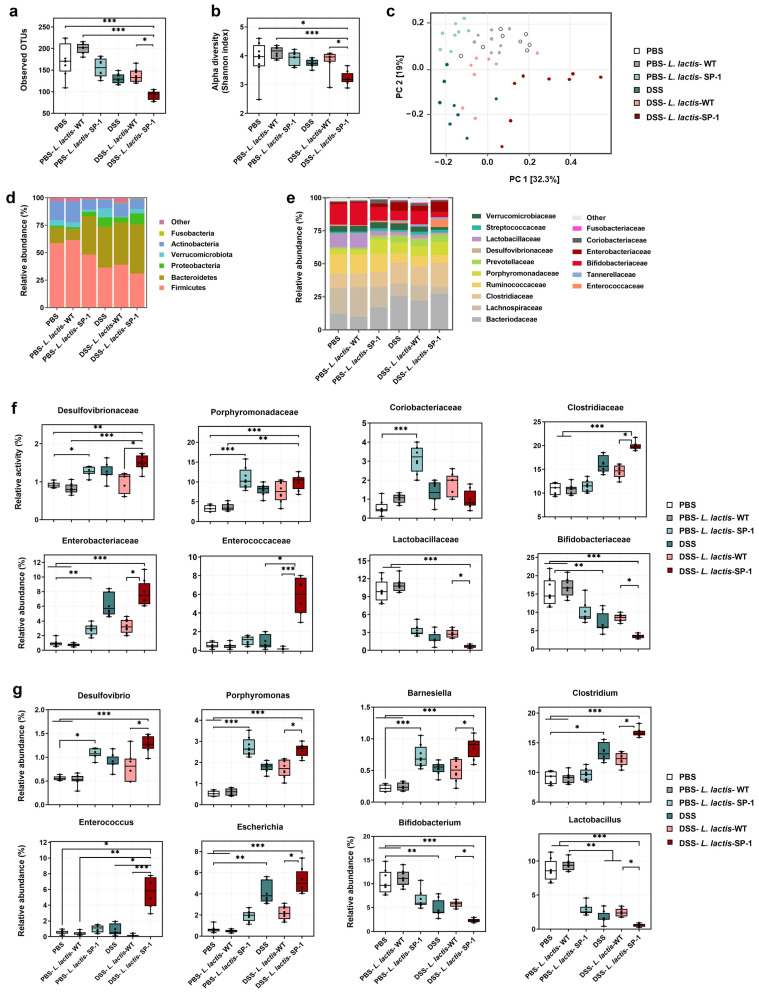
SP-1 administration alters the composition of gut microbiota. (**a**) Estimation of microbial community observed OTU richness and (**b**) α-diversity (Shannon index). (**c**) PCoA plot illustrating the gut microbiota β-diversity. Each point represents each mouse based on a subsample of 12803 OTUs. (**d**–**e**) Relative abundance of gut microbiota. Phylum- and family-level taxonomy are presented as a percentage of total sequences. (**f**) Microbial families with significantly different abundance between studied groups. (**g**) Microbial genera with significantly different abundance between studied groups. Data are presented as mean ± SEM. Data were analyzed by the Kruskal–Wallis test followed by Dunn’s test. * *p* < 0.05; ** *p* < 0.01 and *** *p* < 0.001.

It is well known that epithelial barrier dysfunction and a dysregulated gut microbiota are highly associated with gut inflammation [47,48]. Thus, we examined whether administered *L. lactis* secreting SP-1 altered the composition of gut microbiota in the presence or absence of DSS. 16S rRNA pyrosequencing analysis of fecal samples showed that DSS-*L. lactis*-SP-1 supplementation significantly reduced bacterial richness compared with DSS-*L. lactis*-WT, PBS-*L. lactis*-WT and PBS groups (*p* < 0.001, *p* < 0.05, respectively) (Figure 6a). The Shannon index revealed significant differences in bacterial α-diversity between the DSS-*L. lactis*-SP-1 and DSS-*L. lactis*-WT group (*p* < 0.05) (Figure 6b). Principal coordinate analysis (PCoA) revealed a distinctly different clustering of the gut microbiota between DSS-*L. lactis*-SP-1 and the other groups (Figure 6c). Subsequent analysis at the phylum level revealed that Firmicutes, Bacteroidetes, and Actinobacteria did account for more than 90% of total bacteria in PBS mice (Figure 6d). Of note, PBS-*L. lactis*-SP-1 mice showed increased proportions of Bacteroidetes and Proteobacteria, whereas Verrucomicrobiota remained at a low level (Figure 6d). DSS treatment altered the bacterial structure, thus contributing to increased populations of Proteobacteria and Bacteroidetes and reduced Actinobacteria and Firmicutes *versus* PBS mice (Figure 6d). Interestingly, the administration of DSS-*L. lactis*-SP-1 upheld such phylum-level shift by expanding the abundance of Proteobacteria and Bacteroidetes while limiting the abundance of Actinobacteria and Firmicutes (Figure 6d). At the family level, we found that PBS-*L. lactis*-SP-1 mice exhibited significantly higher Desulfovibrionaceae, Porphyromonadaceae, and Coriobacteriaceae than PBS-*L. lactis*-WT and PBS (Figure 6e–f). In the DSS-*L. lactis*-SP-1 group, increased abundances of Enterococcaceae, Enterobacteriaceae, Desulfovibrionaceae, and Clostridiaceae were observed in comparison to DSS-*L. lactis*-WT (Figure 6e–f). On the other hand, levels of Lactobacillaceae and Bifidobacteriaceae were significantly reduced (Figure 6f).

Further analysis at the genus level showed higher abundances of *Desulfovibrio*, *Porphyromonas*, and *Barnesiella* in PBS-*L. lactis*-SP-1 than PBS-*L. lactis*-WT and PBS mice (Figure 6g). DSS-*L. lactis*-SP-1 exhibited similar alterations that were reported above in the case of PBS-*L. lactis*-SP-1 besides increased levels of *Enterococcus*, *Escherichia*, and *Clostridium* genera (Figure 6g).

**Figure 7:**
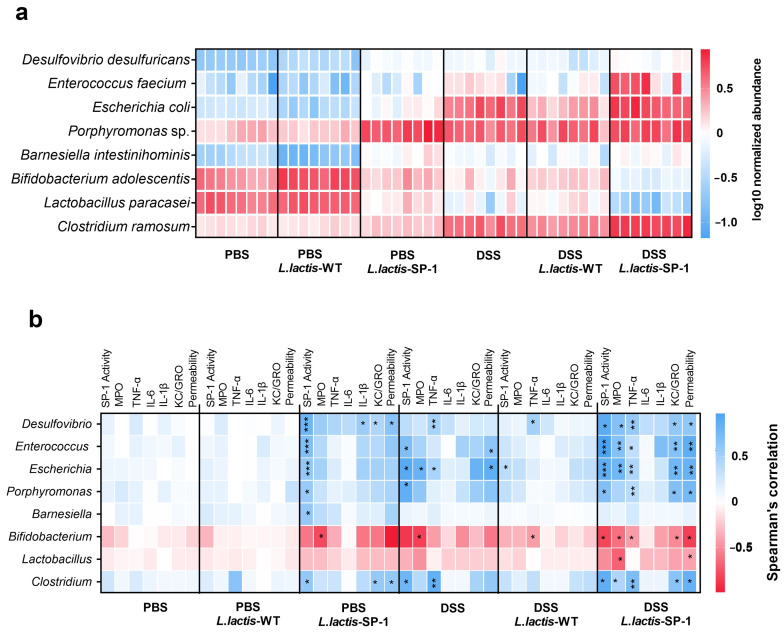
Taxa differentially abundant and correlation with inflammatory parameters. (**a**) Heat map showing the relative abundance (log-transformed) of differentially abundant species between studied groups. (**b**) Spearman correlations between microbial populations and host parameters. The colors denote the nature of the correlation, with dark blue indicating a strong positive correlation and dark red indicating a strong negative correlation.* *p* < 0.05; ** *p* < 0.01; *** *p* < 0.001 after FDR correction.

At the species level, heat map analysis showed that *Porphyromonas* sp., *Enterococcus feacium*, *Escherichia coli,* and *Clostridium ramosum* were significantly abundant in DSS-*L. lactis*-SP-1 (Figure 7a). On the other hand, lower abundances of *Lactobacillus paracasei* and *Bifidobacterium adolescentis* were noted compared with the PBS group (Figure 7a).

To further explore the relationship between the identified taxa and host parameters, we performed multiple correlation analyses. In the PBS-*L. lactis*-SP-1 group, we mainly found positive correlations between SP-1 activity and the bacterial taxa: *Desulfovibrio*, *Enterococcus*, *Escherichia, Barnesiella*, *Porphyromonas,* and *Clostridium* (Figure 7a). In the presence of DSS-*L. lactis*-SP-1, we identified significant positive correlations between *Desulfovibrio*, *Enterococcus*, *Escherichia*, *Porphyromonas*, and *Clostridium* and host physiological parameters including: SP-1 activity, TNF-α, KC/GRO, and intestinal permeability (Figure 7b). Conversely, a negative correlation between intestinal permeability, KC-GRO, TNF-α, MPO, and SP-1 activity with *Bifidobacterium* genus was detected (Figure 7b). We also found a negative correlation between MPO activity and *Lactobacillus* (Figure 7b).

## 4. Discussion

The gut microbiota has become increasingly recognized as a key contributor to the pathogenesis of IBD [47,48,49,50,51]. Clostridia are among the most interesting bacteria in the gut [52]. They have been shown to considerably contribute to the production of colonic serine proteases, and significant alterations in fecal microbiota were detected in patients with IBD [26,27,53]. Previous studies have highlighted increased abundances of some *Clostridium* species, namely *C. ramosum,* in mice with DSS-induced inflammation and IBD patients [9,10]. In this study, we were particularly interested in exploring whether increased microbial protease activity contributes to pathogenic conditions in the gut and potentiates disease. To that aim, we first investigated the serine protease repertoire across *Clostridium* gut species. Our analysis showed that subtilisin-like proteases are likely to be ubiquitous in *Clostridium* and are, in fact, encoded by several species, including those associated with human pathogenicity and disease [54]. Phylogenetic analysis of these sequences revealed a separate clade of uncharacterized subtilisin-like proteins, of which SP-1 from *C. ramosum* did share low sequences identities with the other groups (Figure 1b). To obtain better insights into the role of this protease, the expression and one-step purification of active SP-1 were achieved with sufficient yields for functional analysis. A combinatorial library of protease substrates was then synthesized and screened against SP-1. As indicated, in position P1, both Arg and Lys were preferred over other residues (Figure 3a). Note that most subtilisin-like proteases reportedly do not cleave substrates with basic residues at this position and often show a preference for Glu, Asp, Phe, and Leu [55,56]. This result is, though, in agreement with data provided by Gosalia et al. of a subtilisin Carlsberg accepting P1 basic residues [57]. The presence of non-polar aliphatic residues at positions P4 and P3 were preferred, although other residues were also accepted. With this method, a peptide substrate with the sequence ABZ-Ileu-Met-Ala-Arg-ANB-NH_2_ was finally tailored for SP-1, which showed a higher cleavage efficiency (Table 3). The latter represents an excellent tool to monitor SP-1 activity in biological material, considering that available substrates are never fully specific for one peptidase, a major challenge in studying proteases. Given that increased gut bacterial protease activity has been seldom addressed in GI inflammation, we explored the effects of delivering auxiliary SP-1, using engineered *L. lactis,* on host responses and gut homeostasis. Administration of PBS-*L. lactis*-SP-1 demonstrated relevant signs of inflammation, including weight loss, colon length shortening, and increased IL-6 level. While these changes did not reach statistical significance, they show a consistent signal and suggest that SP-1 could contribute to inflammatory processes rather than inducing inflammation on its own. Of interest, the significant increase in inflammatory parameters observed in this study following DSS treatment was in line with previous data [58] and indicates that the IBD mouse model has been successfully established. In the presence of DSS, we showed that SP-1 inflicted colon and tissue damage, triggered pro-inflammatory cytokine secretion (TNF-α, IL-6, IL-1β, KC/GRO), and exacerbated DSS-induced gut barrier dysfunction. Experimental approaches using Caco-2 cells confirmed that barrier damage was associated with a decrease in TEER in the presence of recombinant SP-1. These findings support a growing body of literature linking intestinal barrier loss to IBD [59,60,61]. As expected, protease activity was significantly higher in DSS-*L. lactis*-SP-1 than DSS-*L. lactis*-WT. Inhibition studies revealed that serine proteases were mainly responsible for the observed activity, which is known to be upregulated in IBD [62]. Additionally, the screening of fecal proteolytic activities using the designed substrate revealed that SP-1 increases the most in DSS-*L. lactis*-SP-1 samples. This is in line with a prior report demonstrating that high fecal protease activity is able to evoke an immediate increase in intestinal permeability [63]. Therefore, we may speculate that the high level of SP-1 in the colonic luminal content of DSS-treated mice is able to trigger permeability and exacerbate mild inflammation in vivo. To assess whether this activity had an impact on gut microbiota composition, we performed 16S rRNA pyrosequencing and highlighted significant alterations resulting from SP-1 administration. A higher abundance of *Desulfovibrio*, an inflammation-related bacterium, was particularly noted in the presence of PBS-*L. lactis-* SP-1, which was further pronounced in DSS-*L. lactis*-SP-1. In the presence of DSS, SP-1 also promoted the expansion of other taxa, mainly *Escherichia* and *Enterococcus*, at the expense of known genera with potent anti-inflammatory properties (Figure 6g). Importantly, we observed increased abundances of *C. ramosum* in DSS and DSS-*L. lactis*-SP-1 microbiota, thus pinpointing a potential contribution to inflammation [64,65]. Higher proportions of *Enterococcus faecium* and *Escherichia coli* were detected as well, thereby suggesting that SP-1 may influence other microbial communities than *C. ramosum*. These findings imply that SP-1 may target the microbiota that shifts from symbiont to potentially pathobiont. Another interesting finding of this study was the identification of significant positive correlations between *Clostridium* abundance, SP-1 activity, and host inflammatory parameters, including intestinal permeability and colonic TNF-α and KC/GRO levels. Significant correlations were also detected between SP-1 activity and pathobionts associated with IBD, such as *Desulfovibrio*, *Enterococcus,* and *Escherichia* in both PBS-*L. lactis*-SP-1 and DSS-*L. lactis*-SP-1 [66,67,68,69]. These findings suggest that the changes observed in the gut environment, in the presence of DSS, are to some extent influenced by SP-1 activity. On the other hand, SP-1 activity correlated negatively with the abundance of *Bifidobacterium* that was shown to exhibit anti-inflammatory properties and reported to be reduced in IBD [70]. In this study, we were also able to correlate several other host parameters, including colonic TNF-α and KC/GRO levels, as well as SP-1 activity with the abundance of other bacterial taxa such as *Porphyromonas,* suggesting that the intestinal dysbiosis driven by SP-1 might be involved in the disease.

**Figure 8:**
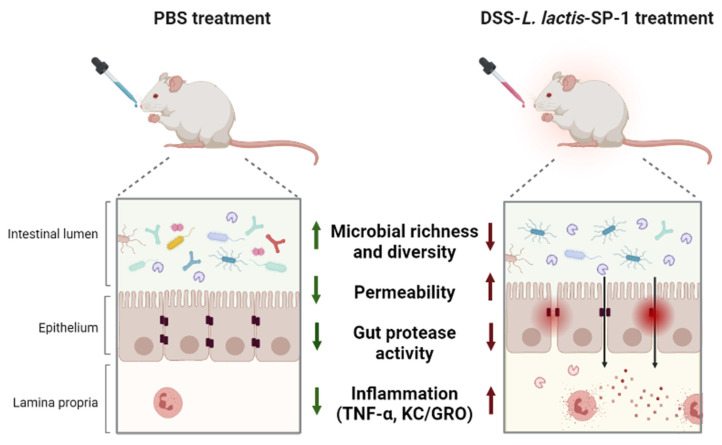
Graphic summary of the study design and main findings.

In summary, this study has (i) reported the functional characterization of a newly identified serine protease from a dysbiotic gut bacterium, and (ii) shown that SP-1 is able to exert potential pro-inflammatory responses during colitis and exacerbate inflammation (Figure 8). For the first time, we showed that these effects occurred with a major impact on the composition of mouse gut microbiota. While SP-1 is probably one of many other factors contributing to gut inflammation, our results clearly raise the question of the relevance of gut protease activities and the mechanisms involved in IBD pathogenesis. Further mechanistic studies (using a specific inhibitor for SP-1) will provide insights into its contribution to the disease.

## Figures and Tables

**Table 1:** Disease activity index (DAI) parameters and their related scoring schemes.

Score	Weight Loss (%)	Stool Consistency	Visible Blood in Stool
0	None	Normal	No bleeding
1	1–5	Loose stools	Slight bleeding
2	5–10		
3	10–15	Diarrhea	Gross bleeding
4	>15		

**Table 2:** Specific substrates.

Substrate	Sequence
SP-1	ABZ-Ileu-Met-Ala-Arg-ANB-NH_2_
Trypsin-like	AB_2_-Val-Val-Ser-Lys-ANB-NH_2_
Elastase-like	AB_2_-Met-Pro-Val-Ala-Trp-Glu-Tyr-(3-NO_2_)-NH_2_
PR3-like	AB_2_-Tyr-Tyr-ABU-Asn-Glu-Pro-Tyr-(3-NO_2_)-NH_2_

**Table 3:** Steady-state kinetic parameters.

Substrate	KM × 10^−4^ (M)	kcat (s^−1^)	kcat/KM × 10^4^ (M^−1^s^−1^)
ABZ-Ileu-Met-Ala-Arg-ANB-NH_2_	1.91 ± 0.3	2.83 ± 0.5	1.48
AB_2_-Val-Val-Ser-Lys-ANB-NH_2_	6.12 ± 0.5	1.79 ± 0.2	0.29
AB_2_-Met-Pro-Val-Ala-Trp-Glu-Tyr-(3-NO_2_)-NH_2_	17.7 ± 0.1	0.46 ± 0.08	0.025
AB_2_-Tyr-Tyr-ABU-Asn-Glu-Pro-Tyr-(3-NO_2_)-NH_2_	14.1 ± 0.7	0.40 ± 0.06	0.027

## Data Availability

Data supporting the findings of this study are available upon request from the corresponding author.
